# The Dignity of the Frail: On Compassion, Terror, and Social Death

**DOI:** 10.1353/lm.2020.0025

**Published:** 2020

**Authors:** Joshua Hordern

**Keywords:** Dignity, frailty, compassion, old age, terror, membership, Psalms, beatitudes, joy

## Abstract

This enquiry considers how the dignity of the frail elderly is objectively grounded, socially constructed, and subjectively experienced. The lives of the frail trouble public consciousness. A terror of old age, felt by young or old, is liable to form a toxic affective culture of social death. Against such threats, the dignity of the frail requires defense. However, empathy- and capacities-based approaches to dignity fail to give a compelling account of humanity’s membership in shared community. By contrast, the poetry of the Psalms and New Testament puts terror to flight by articulating how dignity is found within God’s steadfast, worth-bestowing love which tenderly accompanies humanity in its shared dustiness from the womb to old age and beyond. The blessed dignity these sources describe is found to be more conceptually robust and affectively compelling than an individualistic eudaimonism. Cultivating an ecology of dignity in practice is finally shown to depend on a compassion which grows from the same fertile, imaginative ground.

## Introduction: Frailty, Love, and Terror

This enquiry concerns “the human dignity of the oldest elderly,”^1^ especially the frailest among them: how such “dignity” is grounded, socially constructed, and subjectively experienced. Across many societies, the condition of such people’s lives troubles public and interpersonal consciousness. Publicly, such neuralgic concerns center on decent care for numerous, increasingly long-lived citizens. This has become more pressing amidst risks associated with older age, especially the risks of death and excruciating, long-term illness during the COVID-19 pandemic.

Interpersonally, among younger, healthier people, neuralgia seems linked not only to *relational proximity* to the frail, as children, grandchildren, friends, neighbors, fellow citizens and taxpayers; but also to the *realistic prospect* which the condition of the frailest elderly embody. While they are often, though not always, loved, they may also remind those who care for them about our common frailty. Again, COVID-19 has complicated these relationships, as relational proximity has come into tension with “social distancing” and “self-isolation,” terms now interwoven with the most personal and intimate human relationships.

The well-established distinction between a “Third Age” and “Fourth Age” of human life sets the scene further.^2^ A Third Age of relatively healthy aging has been expanded and extended for many by effective health care and policy changes aimed at minimizing the duration and severity of illness prior to death. According to theologian Frits de Lange, as the Third Age’s length and vibrancy has become increasingly definitive of successful living, so has an awareness of a [Other P-349] decrepit Fourth Age grown as an “embarrassing rest category of the Third Age, a shadowland of diminishment and the portal to death, the result of our inability to eliminate the impairments at the end of life and to push the vital and healthy Third Age until the very moment of death.”^3^

Embarrassment, while important, is not the only relevant affection (or emotion) accompanying people’s understanding of the transition from one age to another. Human affections will vary according to factors such as economic circumstances, culture, and the social construction of gender.^4^ In advanced economies at least, affections which suffuse narrations of the human life-course seem influenced by tendencies towards over-treatment and an emphasis on personal responsibility for health. To the extent that human self-management, biomedical science, and preventive medicine fail to reduce the Fourth Age almost to a vanishing point—to that extent, we are in a narrative of failure. That narrative stands in some contrast, as we shall see, to certain durable literary ways of narrating life, aging, and death, especially those found in the poetry of the Hebrew Bible and New Testament foregrounded here.

Embarrassment about stooping beneath what de Lange calls the “portal to death” should evince more than a shrug of the shoulders. Rather, severe, lingering frailty prior to death constitutes an embodied, existential challenge to a hyper-vigilant health culture, optimistic about technology’s power to master and even abolish human frailty. The Scriptural poetry considered below casts a vision of dignity, set within the imaginative landscape of divine compassion and human worth, which contests such a culture. This matters since, alongside embarrassment about frailty, there may also be a disgust, among those younger or fitter, towards the declining, Fourth-Age elderly. Such elderly may appear as “another class of human beings . . . a different kind of people,” as members of a different world, unknown to the vast majority, and yet imaginably similar to oneself.^5^ This disgust’s strange rationale is, therefore, that such older people represent possible and unwelcome human futures. Such elder-aversion indicates that we “do not love our elderly neighbor as we do not love our aging self.”^6^

Such embarrassment and disgust may become terror. This may take form in passionate, loving concern for the wellbeing of someone for whom one is terrified. However, underneath such terror may lurk a toxic, pathological form, characterizing a love of self with its back to the wall of time’s inexorable advance, no path back to vibrancy and only the grim possibility ahead of decrepit decline. Such terror’s [Other P-350] object, then, is a bleak future of mental and physical diminishment, loss of agency, and the fading memory of youth. Seen in this light, ageism and elder abuse constitute a terror-management strategy, in which fear reduces the cognitive, imaginative space for compassionate love. Anxiety is managed by diminishing those decrepit “others” whom one fears to become like. Although this will not fully explain anxiety and fear of aging, and although terror of death seems to lessen (on average) among the elderly themselves,^7^ and although the reality of frailty may be much better, more hopeful and more joyful than the ghastly decrepitude often dreaded, yet this assessment of the emotional culture around frail old age, formed by the narrative of the Third and Fourth Ages, provides a plausible lens through which to consider the dignity of the frail. While the younger and fitter often are *not* characterized by terror, the assessment indicates that the younger and healthier one is, the more inclined to age anxiety, embarrassment, disgust, and terror one may be.

## Membership

As already intimated, these affective phenomena are interwoven within social and political practice. They interlock especially with questions of *membership*—of who is included within or excluded from the community of persons, a core ethical aspect of dignity to which we shall repeatedly return. Drawing on ethnographic fieldwork, Michael Banner observes that “we have created an old age which for very many is bleak and lonely and during which their social deaths precede their bodily deaths by a number of years. For very many and not just for those suffering from dementia, their experience of aging (which will include debility, dependence, poverty, and marginalization), will itself amount to a death before death, since even without dementia they may well suffer a severe loss of social connections and bonds, and thus a virtual exile from society.”^8^

Allied to this cultural reality of attenuated societal membership is the problematically restricted concept of personhood characteristic of predominant, liberal political theory. This concept is summarized by the political theorist Jeremy Williams and may be best grasped by its implication; namely “that those who remain conscious (or indeed self-conscious), though in a state of dementia or cognitive impairment sufficiently severe to preclude active citizenship and rational project pursuit, can . . . be subsumed into the category of the dead (at least [Other P-351] other things being equal).”^9^ Such a problematic view stems from the Rawlsian notion (which Williams critiques) of “public reason [which] recognises only the rights and interests of persons as legitimate grounds for political action. Thus, for public reason to withhold the status of person from someone is . . . for it to deem them ‘socially dead.’”^10^ Public reason is inclined to withhold the “status of person” from those irretrievably incapable of exercising the power of moral choice—those seemingly not competent to be members of a *deliberative* democracy.^11^ In such a light, terror has a certain reasonableness.

A quite different vision of membership, independent of terror, follows from Hebrew and New Testament poetry:

The Lord is good to all,
  and his compassion is over all that he has made. . .
The Lord upholds all who are falling,
  and raises up all who are bowed down.^12^

In these long-sung expressions of universally available tenderness, to which we will return, there is a vision of dignity which puts terror to flight.

## 1. A Threefold Dignity

Before turning to these sources, it is necessary to consider dignity’s conceptual structure. A threefold distinction within “dignity” provides concepts and terminology necessary in order, in due course, to articulate an ethical and literary response to embarrassment, disgust, and terror regarding the frailest elderly.

First, there is the *objective status* which is called “dignity” in colloquial phrases such as “the dignity of the human person.” To this “dignity,” adjectives such as “inherent” and “inalienable” are often attached. To speak of such dignity invites the question of what, if anything, such a status consists in (e.g. the equal worth of a human person with all other human persons) and how it is grounded (e.g. some metaphysical truth about all human persons). Inasmuch as all who have such a status are united by that which is unchosen by them and so does not require recognition by anyone in order to be true about them, there is an objective basis for determining to whom membership in human community belongs. What requires further specification is an account of who is included in membership and [Other P-352] what ethical obligations will recognize such membership. Whatever this status consists in, however it is grounded and whomsoever is included within it, let us call it “objective dignity.”

Second, there is the *subjective experience* of dignity: of knowing oneself as someone treated by others in ways which accord with *what one takes one’s objective dignity to be*. The contrary is possible of course—the subjective experience of *in*dignity whereby one knows oneself as treated in a fashion which ill accords with what one takes one’s objective dignity to be. Such subjective experience is, therefore, substantially determined by the reckoning one has of what constitutes and accords with one’s own objective dignity. Whatever the quality of the reckoning—including the extent to which it coheres with any, perhaps metaphysical, conception of objective dignity—let us call this experience, “subjective dignity.”

Third, and closely associated with though distinct from subjective dignity, there is the *social recognition* of dignity, understood both individually and culturally. Dignity here paradigmatically involves some overt manifestation, normally through an encounter in which at least two persons interact so as to make *evident* a reckoning concerning the dignity of one or more of the parties. As different reckonings of human dignity interface with one another, such encounters potentially alter—for good or ill—individuals’ subjective experience of their dignity. When more-or-less routinized in practices and conventions of regular forms of meeting (e.g. medical consultations, academic seminars), encounters take on the form of *culture*, whereby members of a community are afforded (or not afforded) recognition which accords with what they take their dignity to require. Let us call that which is communicated concerning the dignity of persons through social recognition, whether individually or in cultures of routinized encounters, “social dignity.”

These distinctions need to be concretized to be grasped. Social recognition of dignity clearly admits of variation: times and cultures differ, for example, over what is appropriate in personal encounter (handshakes—firm, gentle, brief or extended; a bow with hands together; eye contact or not; specific verbal greetings; the naming of each party; courteous and rigorous social distancing, etc). The interaction of social dignity with subjective dignity is particularly important for healthcare precisely because healthcare is an inherently social phenomenon involving numerous routinized encounters in institutions such as the United Kingdom National Health Service (NHS). For example, whether and how people—whether health professionals, patients, or relatives—are identified by their name matters greatly to dignity. To call a 95-year-old [Other P-353] woman by her first name may or may not be a social recognition which accords with her subjective dignity.

Banner’s earlier analysis shows why this matters. Attempts to improve the social dignity of the oldest elderly face the challenge of *social death*. Encounters upon which social dignity depend may be attenuated by terror, funding shortages, or other factors, leading to a corrosive solitariness unmitigated by dignifying company. Within the Fourth Age, people are commonly clinically frail: physically and perhaps mentally diminished and vulnerable to significant and sudden decline. However, their frailty may also be characterized by threats which, if realized, will negatively affect their subjective and social dignity. The frail elderly’s *subjective experience* of dignity may be as paper-thin as their skin may seem, vulnerable to being ripped through by their own or others’ terror.

The Fourth Age is then an age of frailty and an age which threatens terror—terror of present prejudice, neglect, and abuse; terror that these assaults on social and subjective dignity may befall aged loved ones; terror among the relatively young or the (presently) healthy aged at the prospect of future frailty. In such circumstances, there are urgent questions both about what claims regarding objective dignity can do to protect against such toxic terrors and how such claims are communicated.

Turning to at least some forms of literature may help. Considering its power to communicate and form culture and to shape ideas of membership, literature functions in a variety of relevant ways, ranging from the diagnostic to the therapeutic, at times critiquing—at other times promoting—some narrative of aging, adumbration of social or subjective dignity, or concept of objective dignity. Instead of operating at this general level, however, this enquiry will consider specific poetic sources, predominantly from the Hebrew Bible, which have made a lasting contribution both to articulating dignity’s threefold form and to making audible the voices of those who fear or know the *in*dignities to which older age exposes people.

## Points of Clarification

Before doing so, we should note three points of clarification. First, there is a categorical difference which distinguishes objective dignity from subjective and social dignity. Objective dignity is about the fact of the matter concerning the unalterable status of human persons, whereas subjective and social dignity are forged by persons’ various [Other P-354] and changing self-understandings and cultures. Status in “objective dignity” has to do centrally, I shall argue below, with the worth of persons. Such worth generates certain kinds of obligation. But the recognition and realization of dignity in subjective and social experience is more ambivalent, depending upon human psychology, cultural variation, and qualities of interpersonal relationships. When a person suffers some overt circumstance which exposes social recognition of their objective status to negative change—e.g. severe frailty making them vulnerable to abuse—then a test of an individual and a culture arises: whether that person will be treated by others with indignity, perhaps by neglect or direct abuse, or in a manner which so accords with their objective status—and perhaps with what they understand their objective status to be—as to improve the quality of both subjective and social dignity.^13^

Second, an important element of subjective and social dignity is that an *abuser*’s objective dignity may become *occluded from recognition* through their own failure to treat others according to their threefold dignity.^14^ Abusers who obscure others’ dignity are themselves reduced in the process. Where a culture permits this—through disorganization, fear of whistleblowing, underfunding, poor management, or some other factor—all who are in some way implicated are demeaned. For example, when someone or some institution within NHS or social care services fails to recognize a person’s dignity in a way which accords with that person’s objective status—and perhaps what that person understands their objective status to be—all involved, the institution and even the whole political community, is to some extent diminished.

Third, however, one who suffers an assault upon their dignity but resists any diminishment in their own recognition of their (or others’) dignity, may elevate both their own and others’ self-understanding. Their resistance may make apparent their subjective dignity and invite both a reassessment of proper social dignity and the nature of objective dignity.^15^ Strangely, it is in what would otherwise be deeply undignified circumstances that dignity—in its threefold form—may become most manifest.

## 2. Dignity’s Constitution

A core challenge arising from the discussion so far is how to account for an objective basis for membership which will overcome threats of anxiety, embarrassment, terror, disgust, and exclusion. Social dignity and subjective dignity, while highly significant for the protection [Other P-355] of the frail, must ultimately be constituted in relation to objective dignity if they are to bring widespread cultural benefit.

An objective basis for membership which can sustain such a culture of dignified belonging is properly discerned by conceptual analysis but is perhaps most powerfully expressed and widely shared through lyrical literature, shared songs which shape a culture. In what follows, each of three candidates for grounding such membership—empathy, capacities, and love—will be considered through the conceptual illumination afforded by culturally influential forms of Hebrew poetry, especially the Psalms. In the process, an account of compassion, interwoven within this threefold dignity, will emerge.

## Dignity and Empathy

To ask whether empathy could offer an objective basis for membership in a culture of dignity, consider first how each person, younger or older, fit or frail, first appeared in the world and gained some form of social dignity. From this, there is a lesson for how humans fade out or disappear from the world. Thinking of the appearance of the “newone”—the unborn infant—James Mumford articulates the relevant questions for social dignity: “Involving as it does [an] assertion of membership, recognition has a decidedly either-or character. You’re not more or less in within the boundary of concern: either you’re in or you’re out . . . . [G]iven that drawing the line somewhere is nowhere avoidable, the fundamental question we face is always: what are the grounds for inclusion? How are we to decide who should be included within the boundary of concern?”^16^

Empathy is one commonly advocated way of securing and enhancing the otherwise ambivalent and unstable phenomenon of social dignity—in “coming to experience the world as someone else does,” empathy recognizes membership, drawing them into your circle of concern.^17^ Mumford’s critique is that empathy “presupposes [. . . a] kind of hyper-Buberian interpersonal encounter where the other is able to reveal himself and engage fully in dialogue.”^18^ But “even the most minimal degree of empathy presupposes a kind of I-Thou encounter into which the extraordinary encounter between a mother and the being hidden deep within her body cannot be incorporated. Whichever way you look at it, to base ‘who’s in and who’s out’ upon empathy ignores the way human beings appear in the world.”^19^ What is the case with infants is not simply the case with the frailest elderly who [Other P-356] are not hidden *within* another person. And yet the difference separating empathy for an infant from that toward the frail elderly is not one of kind but of degree and emotional complexity, since unavailability to an I-Thou relationship may well increase through a growing hiddenness or withdrawal. Thus, not only but especially due to mental decline, isolation, and loneliness, there may be a growing unavailability for empathic recognition whereby social and subjective dignity could be sustained or enhanced.

The emotional complexity is unlike with the newone. Empathy has an apophatic, inarticulate quality for that never-to-be-returned-to hiddenness associated with the womb-enclosed time immemorial of each person. But encountering the frail elderly, especially those who seem to be disappearing or—as is often said of those with advanced dementia—are “no longer there,” may cause terror because they represent an imaginable future.

If social dignity is to be secured by empathy, empathy must be competent to address at least the phenomena of the disappearing frail and terror. Yet there are good reasons for skepticism about such competence. A social dignity dependent on empathy alone seems an insufficient defense against the vagaries of some individuals’ capacity for repressing their terror and others’ ability or willingness to be available for empathic relationships. Moreover, as Paul Bloom observes, empathy is ill-equipped to think about *cohorts* of frail people and tends to introduce bias to favored sub-populations or individuals.^20^ Bloom’s skepticism about empathy is multifaceted. Concerning literature’s much-vaunted role, Bloom notes “the power of fiction to stir up empathy.” However, precisely because of the variety of intent in literature, empathy in itself is not simply morally good nor a robust buttress for a culture of dignity.^21^ Empathy is underdetermined for the purpose of a *beneficial* social recognition. What is required is *good* (or benevolent) empathy, that which not only intelligently grasps others’ ways of inhabiting bodily life but is well-disposed towards them.^22^ For this reason, dignity needs not mere empathy but a “compassion” that, infused with a specific kind of conceptual objectivity and literary depth, can so inhere within social dignity as to give it an enduringly beneficial form.^23^

Hebrew poetry has made a crucial contribution towards envisaging and sustaining just such compassion. Psalm 71 depicts a compassionate relationship within which *certain kinds* of social and subjective dignity, which are conceptually inextricable from objective dignity, are uniquely available. The Psalmist—probably an aged King David—sings that [Other P-357]

In you, O Lord, do I take refuge;
  let me never be put to shame!^24^

The form of the social relationship in which the Lord and the Psalmist stand—it is *in* the Lord that David takes refuge—suggests that every aspect of David’s dignity is fully defined by the relationship. The peculiar recognition which David sees himself as afforded by the Lord is a special form of social dignity, sustaining a certain kind of subjective dignity. The first-person and second-person singular reverberate repeatedly through the Psalm—again and again, it is “I,” “me,” and “my” in relation with “you” and “your.” The ensuing two verses are characteristic of the whole:

In *your* righteousness deliver *me* and rescue *me*;
  incline *your* ear to *me*, and save *me*!
Be to *me* a rock of refuge,
  to which *I* may continually come;
*you* have given the command to save *me*,
  for *you* are *my* rock and *my* fortress.^25^

However, the nature of this I-you relationship is not adequately captured in terms of *social* or *subjective* dignity. Because of who the Lord is, this particular form of social dignity is directly constituted by *objective* dignity. David continues:

For you, O Lord, are my hope,
  my trust, O Lord, from my youth.
Upon you I have leaned from before my birth;
  you are he who took me from my mother’s womb.^26^

In these words, the Lord’s accompaniment of David as the dependable Creator, an ongoing presence from conception to gestation and a midwife at his birth, is intimately expressed. The thought reflects a wider tradition in Hebrew literature. In Job the focus is similarly upon the Lord as midwife, with all creation participating in womb-like and infant experience.^27^ In Isaiah the Lord’s life-course-long accompaniment underpins the dignity of a whole community, the people of Israel:

Listen to me, O house of Jacob,
  all the remnant of the house of Israel,
who have been borne by me from before your birth,
  carried from the womb;

even to your old age I am he,
  and to gray hairs I will carry you.
I have made, and I will bear;
  I will carry and will save.^28^

Here a continuous line is drawn between interpersonal I-you intimacy in the womb and such intimacy in old age: the people’s dignity is found in being tenderly borne or carried from one end of the life-course to the other. In Psalm 71, David frames a specific threat to an aged individual’s social dignity within this narrative. The Psalm’s opening refers to the “shame” or embarrassment which might come upon David, whereby his dignity would lose social recognition. The threat is that the vicissitudes of old age have exposed even a king to dreadful harms:

Do not cast me off in the time of old age;
  forsake me not when my strength is spent.
For my enemies speak concerning me;
  those who watch for my life consult together
and say, “God has forsaken him;
  pursue and seize him,
  for there is none to deliver him.”^29^

With strength gone, the aged king’s abusers gather round. The aggressors’ ageist sentiment is that he is yesterday’s man. His apparent abandonment by God exposes him to indignity, a fate mirroring the social death of the frail elderly, threatened by the toxic terrors of our own time. Nonetheless, the Psalmist’s hope—confident yet tremulous—is that the God who has borne him from his beginning will not forsake him in the end. Conscious of old age’s dangers, the Psalmist’s song continues to treasure their precious compassionate communion and an active life of wise communication:

So even to old age and gray hairs,
  O God, do not forsake me,
until I proclaim your might to another generation,
  your power to all those to come.^30^

Psalm 71, taken together with other features of Hebrew literature, charts the kind of relational depth required in order that empathy may be replaced by a compassion rooted in that which is stable and enduring, namely the interpersonal relationship between the Lord and the [Other P-359] creatures whom the Lord continually accompanies. As other Hebrew poetry expresses it, this accompaniment offers a secure hiddenness that, when culturally embedded, counters the dangerous form of invisibility to which frailty can subject people. Elsewhere David sings:

Keep me as the apple of your eye;
  hide me in the shadow of your wings.^31^

Here hiddenness is not the toxic concealment of social death. Instead, it is a lifelong form of the maternal, enwombed experience to which Mumford draws attention.^32^ It is in the Lord’s sight, under the Lord’s protection, in the Lord’s presence, so the Psalm suggests, that the individual, surrounded by adversity, is hidden and treasured. Taken together with Psalm 71, the Lord’s temporary midwifery in people’s gestation and birth illuminates the Lord’s permanent maternity in their creation, maturing, decline, and old age. This relationship of accompaniment, lyrically expressed and universally enjoyed, provides a basis for each person’s membership in a common family. Securing a culture of membership between the fit and the frail via interpersonal empathy alone seems implausible by comparison. If we are to combat the ageism, elder abuse, and toxic hiddenness of the frail elderly, and enrich our affective culture, a stronger basis for membership and dignity than empathy is required.

## Dignity and Capacities

Setting empathy aside, let us consider another proposed route to bring stability and definition to a culture of dignity, this time by grounding dignity in characteristically human capacities. This contender is, *prima facie*, more amenable to the Hebrew Bible’s thought-world. Mumford notes how the people of South America, often enslaved by the *conquistadores*, were recognized by Bartolomé de Las Casas as fully human on the basis of their rational nature, especially their capacity for self-government. While deployed in an admirable defense of the vulnerable, Las Casas’ thought accorded conceptually with an antique precedent which distinguishes between such a class of people, characterized by deliberative capacity, and those whom Aristotle considered as essentially belonging to a slave subspecies. So while generally speaking Las Casas’ stance was undoubtedly righteous, “the capacities approach can be used in precisely the opposite way. In short, what some meant [Other P-360] for inclusion others have used to exclude.”^33^ Just as humanity could be divided into those competent or incompetent for self-government, so it could be divided into those without, for example, significant cognitive impairment and a subclass of those with such impairment.

The capacities approach, therefore, undermines the basis for shared membership which could counter a toxic affective culture. And yet it has attractive force. In Kant and some post-Kantian thinkers, the founding of social recognition on *capacities* has an objective basis, namely a capacity for morality. Yet this admits of degrees: persons may have more or less of such a capacity. The extent of their moral freedom is the basis for social recognition. Mumford recalls Maximilian Kolbe, the priest who gave his life for another man in a Nazi death camp, thereby indubitably resisting the diminishment of his and others’ dignity. However, the lesson drawn is that “if my dignity is derived from my capacity for morality it follows that dignity is ‘unequally distributed.’”^34^ This matters for membership, which is a matter of being either in or out, and therefore is not amenable to a measurement of more or less. Thinking of newones, Mumford observes that “to take as a condition for entry something which *does* admit of degrees constitutes a major category-mistake”;^35^ there is a binary quality to membership which cannot be judged on a scale. Moreover, that the growth of rational capacities largely depends on others’ agency (teachers, parents, etc.) presents a serious ethical problem. For “if the manifestation of any given capacity in someone is up *to* us, how could it ever impose an obligation *upon* us?”^36^

I propose that what cannot count as a condition for entry also cannot count as a marker of exit. This matters for the frail elderly, especially but by no means exclusively for those affected by neuro-degenerative conditions. Consider how what is true regarding education is also true regarding health care provision. If it is up to “us,” the relatively young or fit, to control health spending and thus what degree (and what rate of diminution) of rational capacity characterizes the frail, then how can such capacity, to the extent that it is held, impose obligations on us? Since it cannot impose obligations, it cannot shape a culture of dignity, in which obligations necessarily follow a stable pattern.

Mumford rejects an argument for infanticide based on infants’ inability to be self-aware because “if, as we know, a child comes to think of itself as a person only to the extent adults treat it as such, to take the possession of a concept of self as a condition of entry would in practice mean that older members of the human race could reject [Other P-361] younger ones simply by retarding their development.”^37^ Similarly, it is possible that the frail elderly become unable to see themselves as persons, reduced in their subjective dignity, because of abuse, general neglect, or untreated mental decline. Making the condition of membership so closely tied to the behavior of those who will judge inclusion or exclusion is inherently problematic.

This matters for policy. If someone has become frail and withdrawn at least partly on account of inadequate care, to the point of no longer functioning in a characteristically human way, this cannot either license any substantive change in the obligations which all relevant parties have towards that person or, most fundamentally, alter the objective dignity which they have. Stated positively, any praiseworthy institutional culture of social dignity (and consequent experience of subjective dignity) requires others who will reliably recognize people as fellow members of a shared community—*by a standard they have not chosen and cannot remove the conditions for meeting*. Hopes for a culture of dignity cannot be rested on the insecure foundations of capacity recognition. Although the experience of subjective dignity and social dignity may be closely linked to the exercise of capacities, and although the enjoyment of membership may differ according to the capacities any individual holds, inclusion or exclusion from membership on the basis of the relative excellence of human beings’ capacities is morally incoherent. Moreover, by introducing a subclass, it allows for the embarrassment, terror, and disgust which mark cultures which are toxic to dignity.

A different approach is required if such dignity is to be or remain a cultural norm. Hebrew poetry affords a powerful criticism of capacities-based accounts of dignity and, with it, a substantive vision of human dignity which is realistic about human frailty. A central theme of Psalm 90 is the passing life of human persons. Addressing God, the Psalmist sings:

You return man to dust
  and say, “Return, O children of man!”
For a thousand years in your sight
  are but as yesterday when it is past,
  or as a watch in the night.

You sweep them away as with a flood; they are like a dream,
  like grass that is renewed in the morning:
in the morning it flourishes and is renewed;
  in the evening it fades and withers. . . .

The years of our life are seventy,
  or even by reason of strength eighty;
yet their span is but toil and trouble;
  they are soon gone, and we fly away.
Who considers the power of your anger,
  and your wrath according to the fear of you?

So teach us to number our days
  that we may get a heart of wisdom.^38^

Evoking Genesis 3:19—”dust you are and to dust you shall return”—the Psalmist here provides a substantive but realistic basis for dignity. He bases inclusion in membership on a common experience of the two days of dust, one’s beginning and end, separated by the number of one’s days. Even if by “reason of strength” the number of years is eighty rather than seventy, the concern is not with that strength, had by degrees, nor the exact longevity of life. The “heart of wisdom” comes through numbering or recognizing the brevity of one’s days, however many they are—and through understanding that all dust returns to dust. The images of the passing dream and the withering grass underscore the emphasis on humanity’s shared dustiness: on the common, transitory experience among all the “children of man.”

This vision of dignity illuminates a tension in both political theory and clinical practice with respect to the capacity for moral choice. Recall the difficulties first of public reason in determining who counts as “dead” and second, of “social death.” What unites the clinical and the political is “deliberation.” The prevalent clinical norm is that “collaborative deliberation” should mark patient-doctor relationships, with allowances made for patients’ lacking competence for various reasons.^39^ For the liberal political theory Williams critiques, only those able to exercise deliberative moral choice are counted members of the political community. On this view, what is decisive for the dignity of the oldest elderly is their continuing power of choice, in forming projects which they themselves believe will fulfill their lives. The degree of their moral capacity determines their degree of membership. Only by inclusion—whether in person or by proxy—within the community of deliberators can dignity be *experienced* and *made known*.

However, for some frail elderly, a deliberative capacity is somehow “beyond them,” whether temporarily or permanently and whether due to some clinical condition, a general tiredness of life, or some other reason. To exclude such people from membership because they [Other P-363] lack the power of deliberation is to reinstate the same division which demarcated slaves from civilized humankind.^40^ This hardly seems fit for a culture of social dignity in health care, especially as regards the care of the frail elderly, whom we love and yet whose condition many most fear. By contrast, the wisdom which consists in a numbering of days does not require any such active deliberation. Instead, the wisdom necessary for dignity is a simple insight that each member of the human species is dust.

## Dignity and Love

And yet, why does dusty humanity have dignity? With empathy and capacities inadequate to the tasks of grounding membership in a human community and of replacing terror with compassion, the enquiry turns to consider how love is represented in Hebrew poetry. Instead of placing hope for satisfaction in the strength of a person or the length of their days, the hope for humanity lies in the love which formed dust and gives it life and joy amidst affliction. Thus the singer of Psalm 90 praises the everlasting Creator Lord:

Lord, you have been our dwelling place
  in all generations.

Before the mountains were brought forth,
  or ever you had formed the earth and the world,
  from everlasting to everlasting you are God.^41^

Later, the Psalm’s theme turns to a longing for an intimacy with this everlasting Lord.

Satisfy us in the morning with your steadfast love,
  that we may rejoice and be glad all our days.
Make us glad for as many days as you have afflicted us,
  and for as many years as we have seen evil.^42^

Bearing in mind the desire for love, joy, and gladness to remain to the end of one’s days, consider again that dimension of dignity which is constituted by the objective equal worth of all people—“objective dignity.” Nicholas Wolterstorff argues that the best (and ultimately only viable) basis of worth, grounding both duties and claim rights, [Other P-364] is the love of God.^43^ For Wolterstorff, rights are grounded in respect for worth which inheres in an entity “on account of some property it has, some capacity it possesses, some activity it has performed or is performing, some relationship in which it stands.”^44^ Thus the “attempt to show that human rights are grounded in human dignity is [an] attempt to pinpoint some property or relationship whose possession by all human beings gives them all a certain worth—some property or relationship on which worth supervenes.”^45^ Wolterstorff argues that rights must be grounded in human dignity (worth).^46^ The question remains as to how to account for that dignity or worth in the first place. Like Mumford, Wolterstorff rejects a capacities-based grounding of human worth,^47^ thinking especially of the dignity of the frail elderly with dementia,^48^ by reference to the Hebrew Scriptures. Unusually, he holds that the image of God cannot account for such worth since, in referring to that image, the biblical writers “were describing The Human Being . . . [in the sense of] properly formed and properly functioning human beings.”^49^ Instead, what accounts for dignity (and, for Wolterstorff, inherent, natural rights) is the *bestowal* of worth. Such worth does not supervene on a quality which people have by degrees but arises “on account of standing in some relation to the originary good.”^50^

The Psalms cited here provide the literary articulation of this thought in a culturally powerful way. In short, worth is bestowed by God upon humanity conjointly with God’s loving creation of each human person. The bestowal of worth (or “dignity”) does not arise on account of humanity’s capacities, or—a hardly thinkable thought—on their empathy for each other. Rather worth *comes with* the relationship of love which has been created. In this relationship, even and especially amidst frailty, satisfaction by love is paramount, compassion is meaningful and enduring gladness and joy are possible in place of a toxic affective culture.

To avoid misunderstanding, note that this does not render human capacities for agency, including empathic agency, as worth*less*. The Psalmist continues:

Let the favor of the Lord our God be upon us,
  and establish the work of our hands upon us;
  yes, establish the work of our hands!^51^

The real dignity of labor is not finally dependent upon humanity’s own capacities but rather on the Lord’s agency which gives longevity to human work even when the agents themselves return to dust. [Other P-365]

This point reflects the general tendency in Hebrew poetry whereby the dignity-bestowing love of God is set in a specific narrative. There is a literary transition *from* the bestowal of love, coinciding with the act of creation, to a relationship of enduring love, which is in turn responsive to creation’s endemic corruption and manifold frailty. In the Psalmist’s poetic phrasing:

The Lord is gracious and merciful,
  slow to anger and abounding in steadfast love.
The Lord is good to all,
  and his compassion is over all that he has made.^52^

Compassion is the form that steadfast, covenantal love takes amidst the failings and frailties which permeate creation. The note of anger bespeaks a realism about the endemic wrongs of neglect and abuse against those who are vulnerable. Thinking of those afflicted by life’s vicissitudes, the Psalmist continues:

The Lord upholds all who are falling,
  and raises up all who are bowed down.
The eyes of all look to you,
  and you give them their food in due season.
You open your hand,
  satisfying the desire of every living thing.^53^

The extent and exercise of compassionate love is not limited to any particular group of people. Rather, it is said to permeate the frailty of life among *all* those “falling” and “bowed down,” the Fourth-Age elderly among them. While there is a particular covenantal relationship of love with those who sing the Psalms, the Psalm sings of a covenant with all creation. This covenant bestows worth in and with the act of creation and continues to recognize worth by accompanying and satisfying “every living thing.”^54^ The ultimate underpinning, therefore, of dignity is the steadfast love by which God creates and satisfies all living beings, bestowing worth in creation and continually recognizing worth through tender accompaniment whereby daily needs are supplied.

We began with the challenges to dignity from a toxic affective culture which infects perceptions of membership. The poetry of the Psalms bespeaks a worth to human creatures which is grounded in God’s covenantal love, unchosen and supremely dignifying. This worth, [Other P-366] properly recognized, evokes a social dignity which recognizes an ineliminable membership in beloved if dusty human life. Such membership, internalized subjectively and realized in social recognition without any measuring of capacities, can dispel the disgust, anxiety, embarrassment and terror which feeds ageism and elder abuse. Inspiring the Psalms’ lyrical wisdom, a covenantal, creational love underpins the objective dignity of all and raises to recognition those who are bowed down, in their own and others’ eyes, lifting all eyes to look to the One who provides life and food with an open hand.

## 3. Dignity and Blessedness

The Psalmist’s celebration of God’s *satisfaction* of people’s desires throughout all their days raises a further question around dignity, namely what positive societal vision should shape perception of frailty towards the end of one’s days. The language of satisfaction is commonly associated with “flourishing.” However, talk of flourishing is not without its difficulties and may even be in some tension with the dignity of the frail, a tension concerning which New Testament poetry, especially its language of “blessedness” or “beatitude,” provides insight.

To see the nature of the difficulty, consider whether the philosophical tradition known as “eudaimonism” is a problem for dignity. Philosopher Martha Nussbaum offers the following account:

In a eudaimonistic ethical theory, the central question asked by a person is, “How should a human being live?” The answer to that question is the person’s conception of *eudaimonia* or human flourishing, a complete human life. A conception of *eudaimonia* is taken to be inclusive of all to which the agent ascribes intrinsic value: if one can show someone that she has omitted something without which she would not think her life complete, then that is a sufficient argument for the addition of the item in question.^55^

De Lange doubts the wisdom of eudaimonist theory, since it prioritizes the achievement of flourishing through free choice and thus has an inherent bias towards the healthy and middle-aged for whom flourishing is realizable—that is, to whose lives items can be added. Moreover, flourishing seems to sound a note at odds with a common experience of frailty. What, de Lange asks, “does compassion for the elderly mean when they are no longer ‘successfully’ aging but are suffering [Other P-367] from old age”?^56^ To answer this question in terms of the promotion of flourishing may miss the commonsense observation that suffering is not typically understood as a kind of flourishing.

Complementing this concern, Wolterstorff argues that eudaimonism of any sort is antithetical to the task of grounding the rights which accord with dignity (worth): “suppose you have a right against me to the good of my treating you a certain way. Whether or not performing that action would make for greater happiness on my part is simply irrelevant to what I should do. I am to do what you have a right to my doing, period. Rights de-center the agent. Instead of the agent’s happiness determining his action, the worth of the recipient and of those others who will be affected by the action is to determine what the agent does.”^57^ Wolterstorff thinks that eudaimonism—understood here as the promotion of “happiness”—cannot take account of certain goods which consist in “passivities” (ways of being *treated*) as distinct from activities (an active life in accord with virtue). In failing to theorize adequately the good of not being wronged, eudaimonism disqualifies itself from compatibility with rights and thus with dignity, the worth which rights attest.^58^ For present purposes, this analysis applies particularly to those persons with Alzheimer’s upon whom Wolterstorff focuses but, more widely, to any frail person experiencing the Fourth Age, who may be particularly vulnerable to ill treatment and exclusion from a community of concern.

Note, that discounting eudaimonism’s role in understanding dignity is not to discount either the importance of things going well in someone’s subjective dignity amidst frailty or others’ responsible agency for ensuring an appropriate culture of social dignity. As discussed earlier, objective dignity (worth) interlocks with the overall social and subjective experience of how worth is recognized and the active forms of recognition which that worth requires. However, what “going well” amidst adversity means must be integrated with the realities of frailty and the surprising opportunities such experiences can afford. In New Testament literature, those knowing blessedness in life amidst difficulties are the Μακάριοι (makarioi). Their beatitude finds lyrical, Jewish expression in poetry ascribed to Jesus of Nazareth:

Blessed are the poor in spirit,
  for theirs is the kingdom of heaven.
Blessed are those who mourn,
  for they shall be comforted.^59^

These words, taken together with the Psalms considered above, with their notes of gladness and joy amidst adversity, represent a counterpoint to an additive eudaimonism. They suggest that a surprising blessedness can occur amidst hardship, poverty, and mourning. On the one hand, the poor in spirit—those who are “bowed down” in the Psalmist’s phrase—have an ineliminable blessedness in light of the promise in which they participate. Such blessedness, guaranteed in the present by a promised future, depends upon and discloses the permanent, inalienable nature of objective dignity. The logic of the beatitudes is that such human dignity—a dignity of the spirit—is all the more luminous when circumstances would seem otherwise. This logic forms the programmatic literary shape of the gospel in which human suffering will be borne in the indignity of crucifixion. In this narrative, the dignity of God and humanity are united in the poet of the beatitudes, who is presented as one who is bowed down in death to lift all up in resurrection. Such a narrative repeats the earlier refrain concerning how the dignity of the frail can unmask abuse and so resist being defined by such abuse. On the one hand, abusers are reduced in the process of abuse. On the other hand, the dignity of the frail can become all the more apparent amidst abuse, through the gladness, joy, and hope which sustains them. Although such blessedness is real, it in no way licenses abuse. A further beatitude indicates how important justice is for blessedness:

Blessed are those who hunger and thirst for righteousness,
  For they shall be filled.^60^

The possibility of blessedness amidst difficulties signals a wider theme, namely that, since dignity endures whatever frailties are present, frailty in no way implies a necessary loss of human spirit, agency, or insight. The frail can and often are themselves sources of blessing to others. Those who are bowed down will be lifted up and may lift others too. In the recent British context, this thought has become personified through the endeavors of Captain Tom Moore, who gained an international profile by walking a hundred laps of his garden before his hundredth birthday in order to raise funds for a health service stretched by the COVID pandemic. Captain’s Tom’s age and infirmities serve to highlight rather than in any way detract from his modest, hopeful, active and joyful dignity, inspiring the agency of others.

In summary, the beatitudes’ blessedness amidst mournful circumstances is possible when secured in a relationship of kindly comfort and [Other P-369] kingly inheritance.^61^ The frail’s possession of the kingdom of heaven, set in this poetically expressed eschatology, bespeaks an incomparably deeper foundation for dignity and its ethical requirements than either empathy or capacities can provide. The bestowal of worth through God’s steadfast love in creation is fulfilled in the promised future new creation—the kingdom of heaven. Taken together, bestowed worth and the promised kingdom, both founded in God’s love, ground permanent objective dignity whatever the circumstances and yield the possibility of blessedness even amidst the difficulties and indeed sufferings which often accompany old age. The objective flourishing which coincides with both subjective and social dignity is made secure when rooted in God’s love, whereby the stability of human worth and *post-mortem* blessedness is assured not by the capacities of the frail nor by the reliable empathy of the healthy but by God’s promise to hold fast and receive home again those who once departed the womb but are now departing this life. From this fertile imaginative ground a therapeutic ecology for subjective and social dignity may grow.

## Conclusion: Dignity and Compassion

In conclusion, such an ecology will be best secured by the interweaving of dignity with compassion. De Lange asks what compassion means for those suffering from old age. The answer here is that compassion is the affective form of relationships defined by dignity which can transform toxic affective cultures that threaten the frail. By compassion, toxic terror is put to flight, those who are falling are upheld, and the frail become a blessing.

From the literature discussed above, compassion emerges as the form taken by the steadfast, covenantal love which bestows worth (dignity) amidst the failings and frailties which permeate creation. Each aspect of dignity, as discussed here, is in some way relational and thus can be integrated with compassion, an inherently relational aspect of an affective culture. Compassion is apt to achieve coherence between the different aspects of dignity if defined by the self-same steadfast love upon which bestowed worth and beatitude amidst frailty depends. In turn, dignity requires compassion if dignity is to be expressed in tender practice.

First, informed by objective dignity, compassion contradicts any culture in which *in*dignity has taken root and in which anxiety, disgust, and terror have become normalized. In such a culture, the [Other P-370] sense of shared membership which reflects objective dignity is being corroded. Compassion, which grasps the suffering of any individual always in light of their ineliminable bestowed worth, is the preservative against such corrosion. This means compassion cannot accept anyone’s under-valuation of an individual life, even the individual’s own under-valuation. Compassion informed by objective dignity is not acquiescence to individual perception but rather accords with each individual’s ineliminable bestowed worth, rooted in the love of God, whether subjectively recognized or not.^62^

Second, compassion, as a form of consensual, interdependent, intelligent understanding of suffering, fosters shared exploration of what makes for individuals’ subjective dignity, under hard-pressed circumstances.^63^ Compassion attends to the realities which affect a person’s life, especially their own sense of how their life is going and how they have been treated. When informed through careful listening, compassion can create relational strength whereby assaults on an individual’s subjective dignity may be resisted. Such relationships of compassion endure even when, because of mental decline, direct abuse, or “social death,” people become forgetful of their own worth. In cases of very severe frailty and even despair, such compassion can keep subjective dignity alive.^64^

Finally, as to social dignity, an enquiring, steadfast, and sensitive compassion, when culturally widespread, can sustain social recognition between the frail and those whom they encounter, supporting the preservation of subjective dignity. Ultimately, compassion amidst severe cognitive decline sustains both sides of a communicative relationship, daring in love to ensure that a person’s objective dignity continues to be socially recognized in practice and so protected from abuse. Compassion can achieve all this by constantly bringing to societal attention each person’s shared, equal membership in the suffering community of dust, whose dignity remains forever rooted in “steadfast love.”^65^

## NOTES

1 De Lange, *Loving Later Life*, 3.

2 De Lange, *Loving Later Life*, 68–70. For these terms see, classically, Laslett, *A Fresh Map of Life*. See also Baltes and Smith, “New Frontiers”; and, exploring the fourth age as a “social imaginary,” Gilleard and Higgs, “Social Death.”

3 De Lange, *Loving Later Life*, 5.

4 In advanced economies such as Europe, populations are moving towards “a low percentage of young people and a growing percentage of older people” whereas in other contexts (especially Africa), the demographic patterns are rather different (Harper, *How Population Change Will Transform*, 1). As regards gender, the social roles which women are encouraged to fill—and cultural/political contexts in which they live—will impact variously on their health over their life-course (Harper, “Lengthening of Life,” 213). For discussion of affections, health, and politics see Hordern, *Political Affections*.

5 De Lange, *Loving Later Life*, 78; cf. 78–79 for relevant studies of attitudes to old age.

6 De Lange, *Loving Later Life*, 74.

7 Fessler and Navarrete, “Effect of Age.” Recognizing a terror-culture around death does not require concurrence with the evolutionary psychology of terror-management theory. The sources of affections concerning death are highly disputable (cf. Hordern, *Political Affections*, 137–40).

8 Banner, *Ethics of Everyday Life*, 122.

9 Williams, “Death and Consensus Liberalism,” 19.

10 Williams, “Death and Consensus Liberalism,” 28; citing John Rawls, *Political Liberalism*, 33. For further discussion see Hordern, “Challenge of Healthcare.”

11 For analysis of agency in old age, see Gilleard and Higgs, “Aging without Agency.”

12 Psalm 145:9, 14. This citation and all others are from the English Standard Version. I have chosen to replace “mercy” with “compassion” here and when the same text is cited again. On how the two terms relate, see Hordern, “Compassion and Responsibility for Disease.”

13 In classic terms, a failure in *benevolent love* would involve an inaccurate estimation of the objective dignity of the neighbor (O’Donovan, *Problem of Self-Love*, 29–36). Positively stated, a proper estimation would require a “recognition that his dignity and supreme good are the same as mine” (O’Donovan, *Problem of Self-Love*, 113). Benevolent love thus involves treatment in accord with that objective dignity and teleology.

14 Foster, *Human Dignity*, 5.

15 Foster, *Human Dignity*, 15–16.

16 Mumford, *Ethics at the Beginning of Life*, 123–24. See also Mumford, *Vexed*.

17 Bloom, *Against Empathy*, 16.

18 Mumford, *Ethics at the Beginning of Life*, 135. In this connection, Mumford considers Edith Stein’s notion of empathy as a chore to be worked at. For a brief summary of Martin Buber’s analysis of human interactions in terms of “I-Thou” relations, see Mumford, *Ethics at the Beginning of Life*, 31–32.

19 Mumford, *Ethics at the Beginning of Life*, 136.

20 Bloom, *Against Empathy*, 55.

21 Bloom, *Against Empathy*, 48–49.

22 Mumford, *Ethics at the Beginning of Life*, 126–29.

23
Hordern, “What’s Wrong with ‘Compassion’?”

24 Psalm 71:1.

25 Psalm 71:2–3 (italics added).

26 Psalm 71:5–6.

27 For my discussion of Job on this theme, see Hordern, *Compassion in Healthcare*, chapter 6.

28 Isaiah 46:3–4.

29 Psalm 71:9–11.

30 Psalm 71:18.

31 Psalm 17:8.

32 For this image repeated, see also Psalms 36:7, 57:1, 91:1, 91:4.

33 Mumford, *Ethics at the Beginning of Life*, 138.

34 Mumford, *Ethics at the Beginning of Life*, 143, citing and translating Spaemann, “Über den Begriff der Menschenwürde,” 91.

35 Mumford, *Ethics at the Beginning of Life*, 144.

36 Mumford, *Ethics at the Beginning of Life*, 147.

37 Mumford, *Ethics at the Beginning of Life*, 148.

38 Psalm 90:1–6, 10–12.

39 See Elwyn et al., “Collaborative Deliberation.”

40 Mumford, *Ethics at the Beginning of Life*, 144–45, especially concerning Aristotle on *bouleusis*.

41 Psalm 90:1–2.

42 Psalm 90:14–15.

43 Wolterstorff, *Justice*. This move represents an important expansion of language used thus far. For Wolterstorff (and myself), objective human *dignity* and objective human *worth* both cover a conceptually identical domain. However, deploying “worth” to elucidate the nature of *objective dignity* in no way requires abandoning reference to *subjective dignity* or *social dignity*. Indeed, translating these categories into “self-worth” or “social worth” may incur loss—the former being vulnerable to approximation with positive thinking mantras, the latter suggesting individuals’ worth is linked to the value—especially the economic value—which they bring to society.

44 Wolterstorff, *Justice*, 319; for a fuller statement see p. 95.

45 Wolterstorff, *Justice*, 320.

46 Charles Foster similarly affirms that rights—however important as signals of dignity—cannot be foundational: “[if you burrow] beneath the right . . . you will always find a deeper source. Burrow as far as one can go beneath any right worth defending and . . . you will hit dignity” (Foster, *Human Dignity*, 17).

47 Wolterstorff, *Justice*, 331–32. Foster also observes that a capacities-based approach to the image of God will not account for a PVS (persistent vegetative state) patient’s dignity (Foster, *Human Dignity*, 9).

48 Wolterstorff, *Justice*, 330.

49
Wolterstorff, *Justice*, 352

50 Wolterstorff, *Justice*, 355–56.

51 Psalm 90:17.

52 Psalm 145:8–9.

53 Psalm 145:14–16.

54 For an important intra-theological dispute about whether God’s love is indifferent to value (worth), see the criticisms of Anders Nygren by de Lange (*Loving Later Life*, 49), Wolterstorff (*Justice*, 105ff) and O’Donovan (*Problem of Self-Love*, passim). Here, I pursue an argument which unites these three Reformed thinkers over against Nygren’s (ill-conceived) interpretation of *agape*.

55 Nussbaum, *Upheavals of Thought*, 31–32.

56 De Lange, *Loving Later Life*, x.

57 Wolterstorff, *Justice*, 177–78.

58 For one way of reconciling eudaimonism with dignity, see Foster, *Human Dignity*.

59 Matthew 5:3–4.

60 Matthew 5:6.

61 For a sensitive discussion of thriving amidst suffering, see Stump, *Atonement*, 290–336.

62 For detailed discussion of compassion’s nature, see Hordern, *Compassion in Healthcare*, chapters 2–3; Hordern, “Compassion in Primary and Community Care,” 28–30.

63 For discussion of compassion and consent, see Hordern, *Compassion in Healthcare*, 84–88.

64 This point is relevant to debates on assisted suicide; see Callahan, “When Self-Determination Runs Amok.”

65 Psalm 90:14.
